# Improvement of the transient expression system for production of recombinant proteins in plants

**DOI:** 10.1038/s41598-018-23024-y

**Published:** 2018-03-19

**Authors:** Tsuyoshi Yamamoto, Ken Hoshikawa, Kentaro Ezura, Risa Okazawa, Satoshi Fujita, Miyo Takaoka, Hugh S. Mason, Hiroshi Ezura, Kenji Miura

**Affiliations:** 1 Graduate School of Life and Environmental Sciences, University of Tsukuba, Tsukuba, 305-8572 Japan; 2 Center for Immunotherapy, Vaccines and Virotherapy (CIVV), The Biodesign Institute at Arizona State University, Tempe, AZ 85287 USA

## Abstract

An efficient and high yielding expression system is required to produce recombinant proteins. Furthermore, the transient expression system can be used to identify the localization of proteins in plant cells. In this study, we demonstrated that combination of a geminiviral replication and a double terminator dramatically enhanced the transient protein expression level in plants. The GFP protein was expressed transiently in lettuce, *Nicotiana benthamiana*, tomatoes, eggplants, hot peppers, melons, and orchids with agroinfiltration. Compared to a single terminator, a double terminator enhanced the expression level. A heat shock protein terminator combined with an extensin terminator resulted in the highest protein expression. Transiently expressed GFP was confirmed by immunoblot analysis with anti-GFP antibodies. Quantitative analysis revealed that the geminiviral vector with a double terminator resulted in the expression of at least 3.7 mg/g fresh weight of GFP in *Nicotiana benthamiana*, approximately 2-fold that of the geminiviral vector with a single terminator. These results indicated that combination of the geminiviral replication and a double terminator is a useful tool for transient expression of the gene of interest in plant cells.

## Introduction

Transgenic plants are generally used to obtain recombinant proteins or identify the localization of proteins. However, substantial time is required to generate transgenic plants, and the yield of the expressed protein is relatively low. Alternatively, transient expression systems with virus-based vectors have the advantage of rapid and high-level expression of the recombinant proteins. The replication system of plant viruses results in high-level expression of foreign proteins within a few days^1^. Thus, plant virus expression vectors are attractive, and the production level of the gene of interest can be easily amplified by increasing the number of host plants^2^. A tobamovirus (TMV)-based deconstructed viral system (magnICON) has been extensively engineered to achieve high levels of recombinant protein accumulation in tobacco leaves^3^. ZMapp for *Ebolavirus* infections, which was used during a recent outbreak, was also produced by the magnICON system^4^. Thus, the magnICON system is very useful system in tobacco, including *Nicotiana benthamiana*. However, the system may not be applicable to lettuce leaves^5^. Because of TMV-based viral vector, host factors from a plant species must recognize and interact with TMV elements or factors.

Bean yellow dwarf virus (BeYDV), a *Mastrevirus* of the Geminiviridae family, contains a single-stranded circular DNA genome and uses a rolling circle mechanism to replicate its genome, resulting in a very high yield of copies. This mechanism has been used for boosting protein expression in transgenic plants^6^ and for transient expression of foreign proteins with efficiency in *N. benthamiana* leaves^7^ and in lettuce leaves^5^. Some geminivirus species can replicate in non-host plant cells and BeYDV has a broad host range in dicotyledonous plants^8^. A recent study demonstrated that introduction of PsaK 5′-UTR, extensin (Ext) terminator, and the tobacco Rb7 matrix attachment region into the geminivirus replicon vector greatly improved the expression level of protein with the *Agrobacterium* strain EHA105^9^. The Ext terminator enhanced protein production, compared to *vspB* terminator^9^.

Previous research has demonstrated that the terminator of the heat shock protein (HSP) gene increased the gene expression level in *Arabidopsis* plants^10^, tomatoes^11^, and lettuce^12^. Furthermore, the expression level of the gene was increased with double transcription terminators, the *CaMV 35S* terminator and *NOS* terminator^13,14^; however, these results were obtained from stable transformants. The introduction of a second terminator possibly detects read-through transcripts and traps it by the hairpin structure^15^. Read-through transcripts may cause inhibition of 3′-end cleavage/polyadenylation processing. The less efficient polyadenylation of mRNA leads to reduction of translatable mRNAs and, consequently, decrease in protein production^16,17^. In this study, combining the geminiviral replication system with a double terminator increased the transient protein expression level. In particular, when the HSP and Ext terminators were used as a double terminator, the expression level was the highest and reached approximately 3.7 mg/g fresh weight (FW) in *N. benthamiana*. Furthermore, this system could be applicable to not only *N. benthamiana*, but also tomatoes, eggplants, lettuce, hot peppers, melons, and orchids. These results indicated that this system is a useful tool, with which to express specific proteins in plant cells.

## Results

### A comparison of the expression level of GFP between pBYR2HS and pBYR2fp geminiviral replicon vector

**Figure 1:**
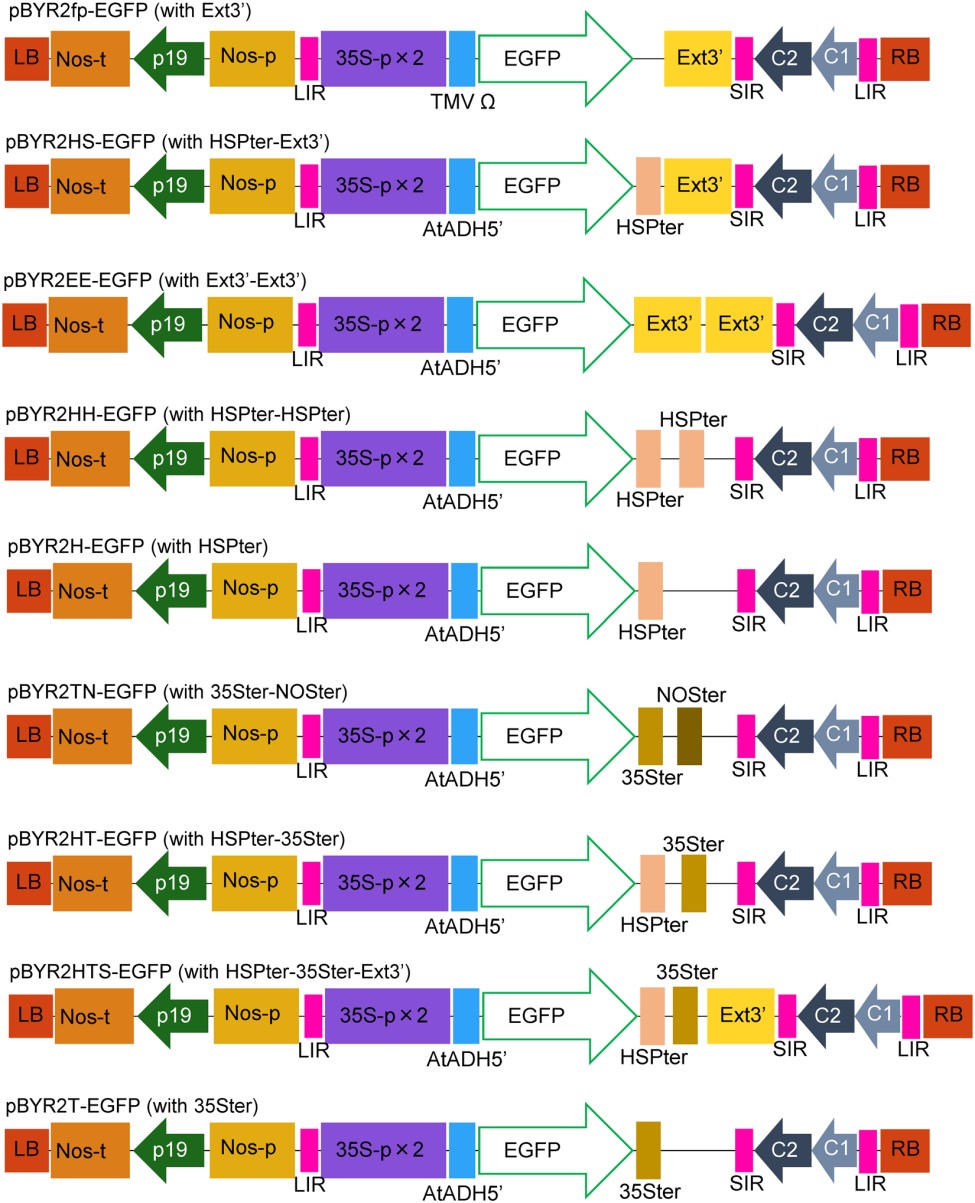
Schematic diagram of the T-DNA region of the plasmids pBYR2fp-EGFP, pBYR2HS-EGFP, pBYR2EE-EGFP, pBYR2HH-EGFP, pBYR2H-EGFP, pBYR2TN-EGFP, pBYR2HT-EGFP, pBYR2HTS-EGFP, and pBYR2T-EGFP. 35S-p x 2, CaMV 35 S promoter with double-enhanced element; AtADH5′, 5′-untranslated region (UTR) of *Arabidopsis thaliana* alcohol dehydrogenase gene; TMV Ω, 5′-leader sequence of tobacco mosaic virus; EGFP, enhanced green fluorescence protein; HSPter, terminator of heat shock protein gene; Ext3′, tobacco extension gene 3′ element; 35Ster, terminator of CaMV 35S; NOSter, NOS terminator; LIR, long intergenic region of bean yellow dwarf virus (BeYDV) genome; SIR, short intergenic region of BeYDV genome; C1/C2, BeYDV ORFs C1 and C2 encoding for replication initiation protein (Rep) and RepA, respectively; LB and RB, the left and right borders of the T-DNA region, respectively; Nos-p and Nos-t, NOS promoter and terminator, respectively; and p19, a gene-silencing suppressor gene from tomato bushy stunt virus.

**Figure 2:**
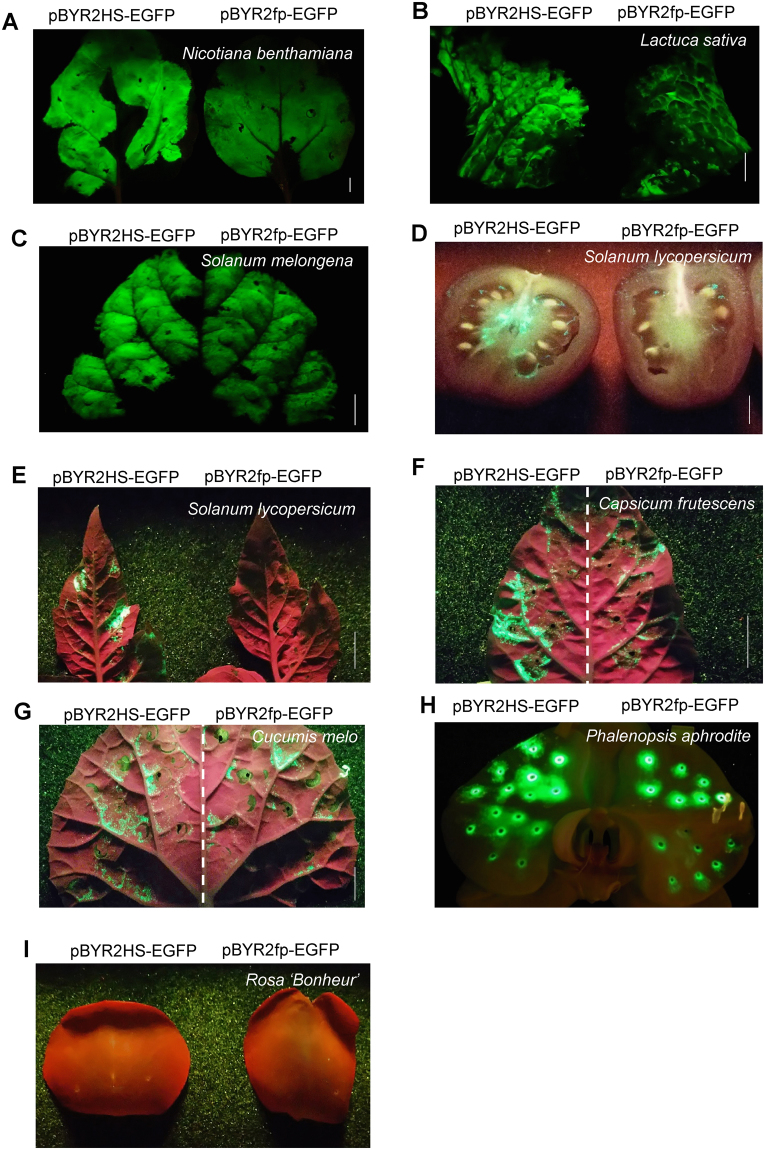
Introduction of HSP terminator improved transient expression of EGFP. *Agrobacterium tumefaciens* harboring pBYR2HS-EGFP and pBYR2fp-EGFP were transfected into *Nicotiana benthamiana* (**A**), lettuce *Lactuca sativa* var. *crispa* (**B**), eggplant *Solanum melongena* cv. ‘Dewakonasu’ (**C**), tomato fruits *Solanum lycopersicum* cv. ‘M82’ (**D**), tomato leaves *Solanum lycopersicum* cv. ‘Micro-Tom’ (**E**), hot pepper *Capsicum frutescens* cv. ‘Shima-togarashi’ (**F**), melon *Cucumis melo* cv. ‘Earl’s Favorite Harukei No.3′ (**G**), orchid *Phalaenopsis Aphrodite* (**H**), and a rose *Rosa* sp. ‘Bonheur’ (**I**). These plants were incubated for 3 days after agroinfiltration. Then, after blue-light excitation, GFP emission was observed with an ultraviolet-absorbing filter Fujifilm SC-52. Bars indicate a 1-cm length.

In the pBYR2HS vector, the alcohol dehydrogenase (AtADH) 5′-UTR region was replaced with tobacco mosaic virus (TMV) Ω and the HSP terminator was inserted into pBYR2fp, resulting in a double terminator construct (Fig. 1). Each vector was transformed into *Agrobacterium tumefaciens* GV3101 and green fluorescent protein (GFP) was transiently expressed in *N. benthamiana* (Fig. 2A), lettuce *Lactuca sativa* (Fig. 2B), eggplants *Solanum melongena* (Fig. 2C), tomato Solanum lycopersicum fruits (Fig. 2D), tomato leaves (Fig. 2E), hot peppers *Capsicum frutescens* (Fig. 2F), melons *Cucumis melo* (Fig. 2G), orchids *Phalaenopsis aphrodite* (Fig. 2H), and roses (Fig. 2I). Transfection with pBYR2HS-EGFP improved expression of GFP in these plants except for the rose, compared with pBYR2fp-EGFP. In particular, GFP fluorescence emission was only observed in tomato fruits and leaves agroinfiltrated with pBYR2HS-EGFP (Fig. 2D,E). No fluorescence was detected in the rose (Fig. 2I).

**Figure 3:**
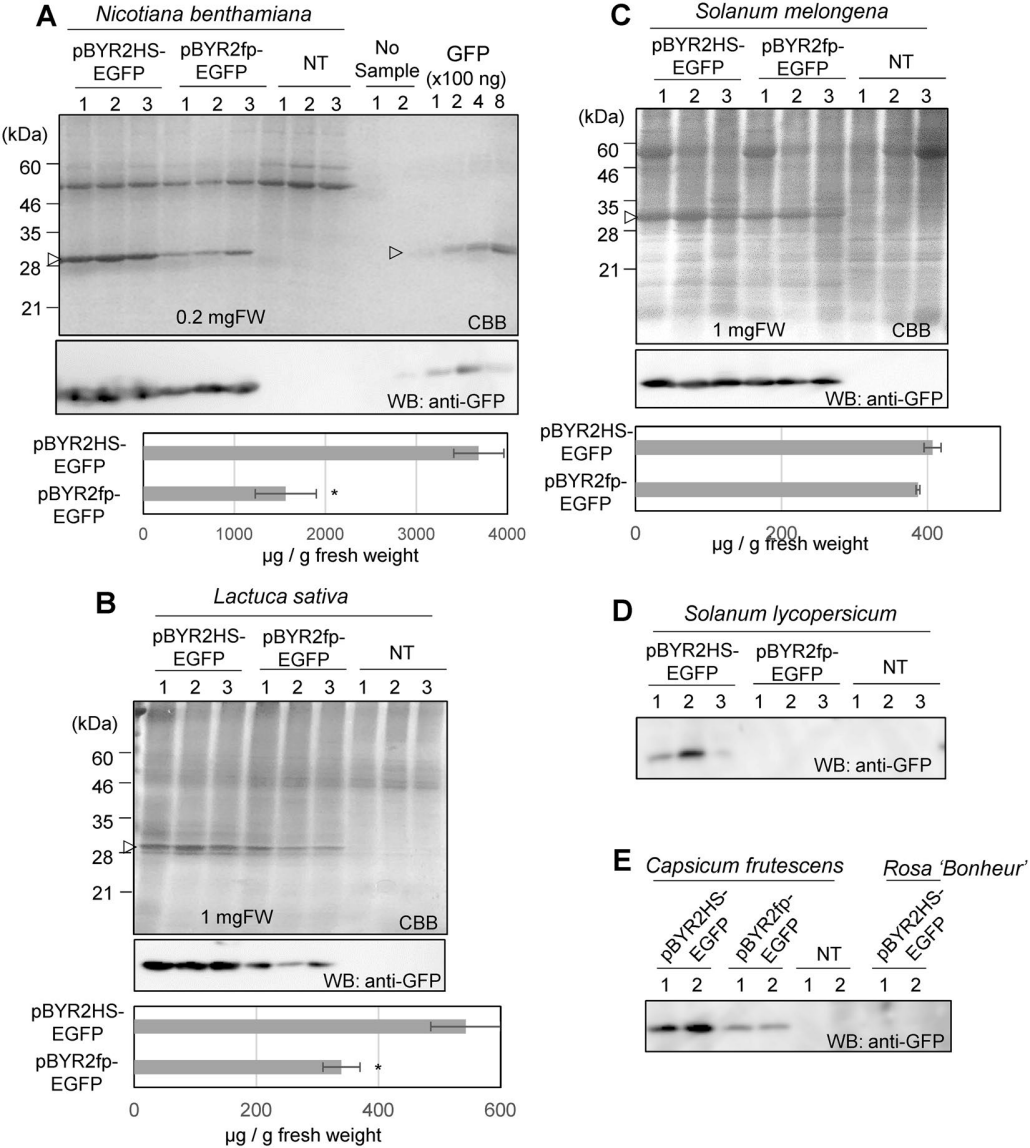
Effect of HSP terminator on transient GFP expression at 3 days post-agroinfiltration. Total soluble proteins were extracted from agroinfiltrated plant leaves with pBYR2HS-EGFP or pBYR2fp-EGFP. Coomassie Brilliant Blue (CBB) staining and immunoblot analysis with anti-GFP antibodies were performed by using agroinfiltrated leaves of *N. benthamiana* (**A**), lettuce (**B**), eggplants (**C**), tomatoes **(D)**, hot peppers, and roses (**E**). The numbers at the top of the gels indicate different samples taken from different leaves from different plants. NT indicates non-transfected plants. The amount of protein was measured according to band intensity from CBB staining gel using ImageJ software. Arrowheads indicate bands corresponding to GFP protein. The band clearly seen at 55 kDa in a CBB staining gel is corresponding to large subunit of Rubisco. Data represent the means ± SD (*n = *3 to 4). Significance was determined using unpaired Student’s *t* tests (*p < 0.05). Full-length gels and full-length blots are presented in Supplementary Figures S1 and S2.

Then, total soluble proteins were prepared from 0.2 mg fresh weight (FW) of *N. benthamiana* and 1 mg FW of lettuce, eggplant, tomato, hot pepper, and rose. The total soluble proteins were detected with Coomassie Brilliant Blue (CBB) staining. The GFP was also detected by immunoblot analysis with anti-GFP antibodies. Expression levels of GFP from plants agroinfiltrated with pBYR2HS-EGFP were higher than that from plants agroinfiltrated with pBYR2fp-EGFP (Fig. 3). Calculation of expression level by image analyzer indicated that approximately 3.7 mg of GFP was expressed from 1 g FW (fresh weight) in *N. benthamiana* agroinfiltrated with pBYR2HS-EGFP (Fig. 3A). Conversely, approximately 1.5 mg of GFP was expressed in 1 g FW in *N. benthamiana* agroinfiltrated with pBYR2fp-EGFP. Similarly, the expression level of GFP from lettuce or eggplant agroinfiltrated with pBYR2HS-EGFP (0.37 mg/g FW or 0.46 mg/g FW, respectively) was higher than that of lettuce or eggplant agroinfiltrated with pBYR2fp-EGFP (0.20 mg/g FW or 0.42 mg/g FW, respectively, Fig. 3B,C). Because a clear GFP band with CBB staining was not observed in tomatoes, hot peppers, and roses, western blot analyses were performed. GFP expression levels of tomato leaves and hot pepper leaves agroinfiltrated with pBYR2HS-EGFP was also increased (Fig. 3D,E). GFP expression in roses agroinfiltrated with pBYR2HS-EGFP was not detected even when the western blot analysis was performed. These results indicated that introduction of the HSP terminator improved the expression level of GFP in the agroinfiltrated plants and this system can be used for several species.

### A double terminator with geminiviral replication enhances transient protein expression

**Figure 4:**
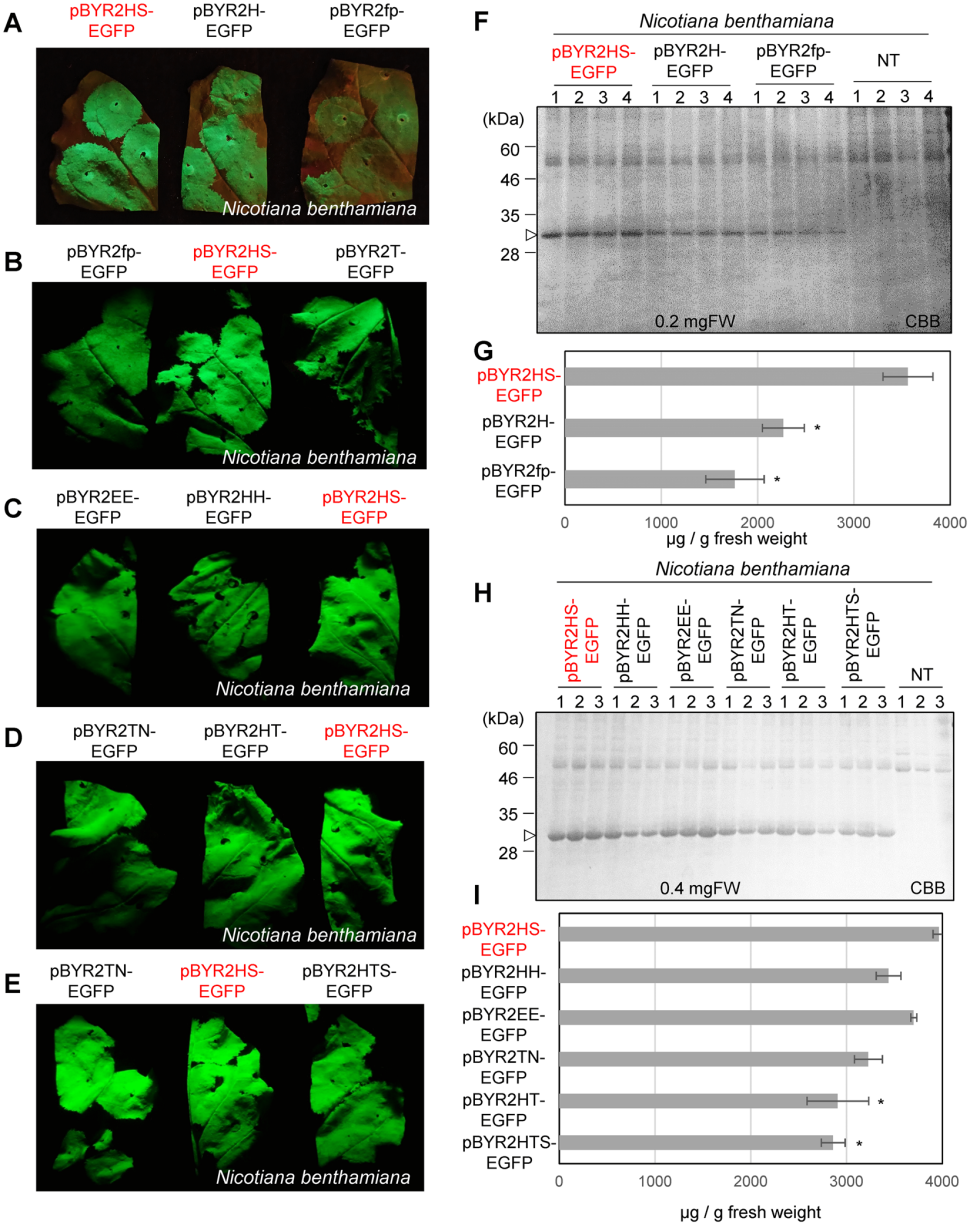
Effect of single or double terminator on transient EGFP expression at 3 days post-agroinfiltration. GFP emission from leaves of *N. benthamiana* agroinfiltrated with several kinds of plasmids (Fig. 1) was observed with an ultraviolet-absorbing filter, Fujifilm SC-52 (**A**–**E**). Total soluble proteins were extracted from agroinfiltrated *N. benthamiana* leaves with pBYR2HS-EGFP, pBYR2H-EGFP, or pBYR2fp-EGFP. Coomassie Brilliant Blue staining were performed (**F**) and the amount of protein was measured (**G**). Total soluble proteins were extracted from agroinfiltrated *N. benthamiana* leaves with pBYR2HS-EGFP, pBYR2HH-EGFP, pBYR2EE-EGFP, pBYR2TN-EGFP, pBYR2HT-EGFP, and pBYR2HTS-EGFP. Coomassie Brilliant Blue staining was performed (**H**) and the amount of protein was measured (**I**). The numbers at the top of the gels indicate different samples taken from different leaves from different plants. Data represent the means ± SD (*n = *3 to 4). Significance was determined using unpaired Student’s *t* tests (*p < 0.05). Full-length gels are presented in Supplementary Figures S3 and S4.

To confirm whether the HSP terminator improved expression of the protein or double terminator enhanced expression, several kinds of plasmids were prepared (Fig. 1). First, pBYR2H-EGFP containing only the HSP terminator, pBYR2fp-EGFP containing only Ext 3′, and pBYR2HS-EGFP containing both the HSP terminator and Ext 3′ were compared. These plasmids were used for agroinfiltration into *N. benthamiana* and the expression of GFP was compared (Fig. 4A,F,G). The expression level of GFP from *N. benthamiana* with pBYR2H-EGFP (2.2 mg/g FW, Fig. 4G) was higher than with pBYR2fp-EGFP (1.7 mg/g FW, Fig. 4G), suggesting that the HSP terminator enhanced expression of GFP. However, if the plasmid contained both the HSP terminator and Ext 3′, the expression level was significantly higher than with plasmids containing the single terminator. Next, several combinations of double terminators were investigated (Fig. 4B–E). In conclusion, the expression level of GFP with pBYR2HS-EGFP (3.9 mg/g FW) was the higher than that obtained with other plasmids, such as pBYR2HH-EGFP (3.4 mg/g FW), pBYR2EE-EGFP (3.7 mg/g FW), pBYR2TN-EGFP (3.2 mg/g FW), pBYR2HT-EGFP (2.9 mg/g FW), and pBYR2HTS-EGFP (2.9 mg/g FW) (Fig. 4H,I). Interestingly, the expression level of GFP with pBYR2HTS-EGFP was statistically lower than that with pBYR2HS-EGFP (Fig. 4I). This suggests that triple terminators may decrease expression of the protein, compared with a double terminator. Taken together that expression level with pBYR2HH-EGFP was slightly lower than that with pBYR2EE-EGFP, it is plausible that too much terminator activity may decrease the protein expression level. These results indicated that the double terminator is sufficient to enhance expression of the protein, and a combination of HSP terminator and Ext 3′ was the best in this experiment.

**Figure 5:**
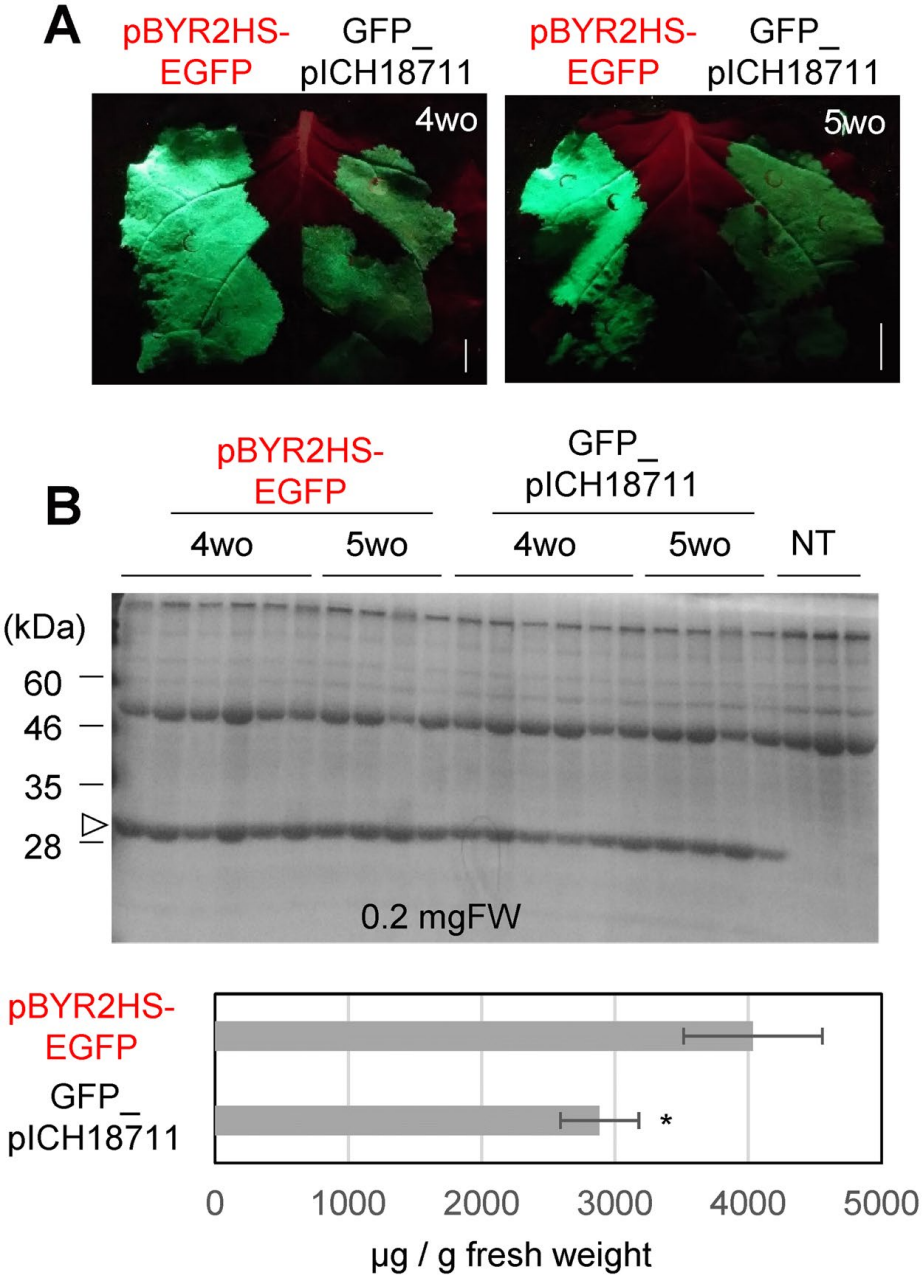
Comparison of expression levels at 3 days post-agroinfiltration. pBYR2HS-EGFP or GFP_pICH18711 was agroinfiltrated into the same leaves of 4-week-old (4wo) or 5-week-old (5wo) *N. benthamiana*, side by side. (**A**) GFP emission was observed. (**B**) Total soluble proteins were extracted from agroinfiltrated *N. benthamiana* leaves. Coomassie Brilliant Blue (CBB) staining were performed. Arrowheads indicate bands corresponding to GFP protein. (**C**) The amount of GFP protein from 4-week-old leaves, which were incubated 3 days after agroinfiltration, was measured according to band intensity from CBB staining gel using ImageJ software. The numbers at the top of the gels indicate different samples taken from different leaves from different plants. Data represent the means ± SD (*n = *6). Significance was determined using unpaired Student’s *t* tests (*p < 0.05). Full-length gels and full-length blots are presented in Supplementary Figure S6.

To examine whether this expression system works well, the system was compared to the magnICON system as a standard viral expression system. Both vectors were agroinfiltrated into the same leaf, side by side. After 3-day incubation, the expression of GFP was compared (Fig. 5A) and total soluble protein was separated with SDS-PAGE (Fig. 5B). The expression level of GFP from 4-week-old *N. benthamiana* with pBYR2HS-EGFP (4.0 mg/g FW, 36% TSP (total soluble protein), Fig. 5C) was significantly higher than that with GFP_pICH18711 (2.9 mg/g FW, 28% TSP, Fig. 5C). These results indicate that protein expression level with our system is higher than that with the magnICON system under our conditions, i.e. 3-day incubation at 25 °C with 16 h light and 8 h dark.

## Discussion

The HSP terminator with the extensin terminator strongly increased protein expression levels in lettuce, *N. benthamiana*, tomatoes, eggplants, hot peppers, and orchids *P. aphrodite*.

Generally, it takes substantial time to produce stable transgenic plants for production of a recombinant protein. Post-transcriptional gene silencing (PTGS) often occurs in plants to reduce the amount of protein expression. PTGS is a natural defense mechanism relying on RNA interference for plants against viruses and pathogenes^18^. Production of excessive RNA transcripts over a threshold level, such as overexpressing a transgene under CaMV 35S promoter, causes transgene silencing through activation of PTGS^19–21^. Thus, the yield of production of several kinds of recombinant proteins in plants is limited by PTGS. Conversely, transient expression in plant leaves solves these problems. After 3 to 7 days of agroinfiltration, recombinant proteins are produced. And when the vacuum-infiltration method is applied, large-scale production of recombinant proteins is possible.

In a previous study, the expression level of the gene was increased with double transcription terminators, *CaMV 35S* and *NOS*^13,14^. The double terminator was useful for the transient expression system (Fig. 4), and a combination of the HSP terminator and Ext terminator was the best in this study (Fig. 4I). For convenience, EGFP sequences were removed from pBYR2HS-EGFP and the *Sal*I site was introduced between AtADH 5′ and the HSP terminator. The resulting vector was named pBYR2HS-SalI and a gene of interest could be introduced into pBYR2HS digested with *Sal*I.

The results indicate that at least 3.7 mg of GFP per 1 g FW of leaf of *N. benthamiana* was able to be produced. To produce proteins in *N. benthamiana*, the magnICON expression system is often used. For example, yields of the *Plasmodium yoelii* merozoite surface protein 4/5, Norwalk virus-like particles, and hepatitis B virus core antigen virus-like particles were 1–2 mg/g FW^22^, 0.8 mg/g FW^23^, and 2.4 mg/g FW^24^, respectively. The magnICON system is shown to be capable of yield up to 4 mg/g FW of GFP^25^. Expression levels of GFP in our system were similar to those in the magnICON system. Under our conditions, i.e. 3-day incubation at 25°C with 16 h light and 8 h dark, protein expression level from *N. benthamiana* leaves agroinfiltrated with the magnICON vector was less than that with pBYR2HS-EGFP (Fig. 5). According to the paper^25^, the peak of GFP expression was at 6 to 10 days post-infiltration and expression level was not very high at 3 days post-infiltration. Thus, protein expression level with the magnICON system was not high under our conditions and the pBYR2HS system may accumulate protein in shorter periods. A recent study demonstrated that changing the 5′ and 3′ untranslated region in the geminivirus replicon system (pBYR2eP3) enhanced expression levels of a protein in *N. benthamiana* and the Norwalk virus capsid protein was produced at 1.8 mg/g FW^9^. It is possible that the introduction of the HSP terminator into pBYR2eP3 for the double terminator further enhances protein production.

Not only the magnICON system, but also several other systems, such as the *E. coli* expression system, *Brevibacillus* expression system, and the baculovirus-insect cell expression system, are available for production of recombinant proteins. Although a simple comparison is not accurate, the amount of protein production is compared as mL of liquid culture equivalent to 1 g. In the *Brevibacillus* expression system, yields of α-amylase, cyclodextrin glucanotransferase, human protein disulfide isomerase, and human epidermal growth factor were 3.7 mg/mL, 1.5 mg/mL, 1.0 mg/mL, and 1.5 mg/mL, with production based on the manufacture’s instruction^26–28^. The yield of GFP-ELP (elastin-like polypeptides) was 1.6 mg/mL in the *E. coli* expression system^29^. The maximum amount of GFPuv was 6.9 mg/g larvae mass with the baculovirus expression system^30^. These results suggest that our system is comparable to other protein expression systems in terms of the quantity of protein expression. The advantage of using plants is the ease of obtaining large quantities of plants at low cost, and no risk of contamination with retroviruses when compared to mammalian cell cultures.

Furthermore, geminivirus replicon has advantages because this replicon system can work in several species (Fig. 2). It is probably because geminivirus has a broad host range^31^. On the other hand, the magnICON system can primarily work in tobacco^32^. As shown in the previous article, the magnICON system may not be used in lettuce^5^. The geminiviral replicon system can be used for not only protein expression, but also determining the localization of the protein of interest in several species.

## Methods

### Vector construction

The pBYR2fp vector harbored a replication system from BeYDV^33^. Furthermore, a p19 RNAi suppressor expression cassette was included in this vector (Fig. 1). To compare expression levels, EGFP was amplified with the primers, pBYR2fp-EGFP-F and EGFP-pBYR2fp-R (Table S1), and the PCR product was inserted into *Xba*I-digested pBYR2fp with an In-Fusion HD Cloning Kit (Takara Bio).

To obtain higher yield of recombinant proteins, 5′-UTR of alcohol dehydrogenase gene and the terminator of heat shock protein, which enhanced gene expression^10,34^, was introduced. EGFP was amplified with the primers, pRI201-EGFP-F and EGFP-pRI201-R (Table S1). The PCR product was inserted into *Nde*I and *Sal*I-digested pRI201-AN (Takara Bio). The resulting vector, pRI201-EGFP, was used as a template for amplification of the cassette (AtADH 5′-UTR-EGFP-HSP terminator) with the primers, pBYR2fp-AtADH-F and pBYR2fp-HSPter-R (Table S1). The cassette was introduced into *Xho*I and *Xba*I-digested pBYR2fp, and pBYR2HS-EGFP was constructed (Fig. 1).

Ext3′ was amplified with the primers, pBYR2EE-Ext3-F and pBYR2EE-Ext3-R (Table S1). The PCR product was inserted into *Sal*I and *XbaI*-digested pBYR2HS-EGFP, resulting in the plasmid pBYR2EE-EGFP (Fig. 1).

To produce pBYR2H-EGFP, Ext3′-SIR-C2 sequences were removed from pBYR2HS-EGFP with *Xma*I and *Cla*I. SIR-C2 sequences were amplified with the primers, HSPter-SIR-F and C1-ClaI-C2-R (Table S1) and inserted into *Xma*I and *Cla*I-digested pBYR2HS-EGFP, resulting in the plasmid pBYR2H-EGFP (Fig. 1). HSPter was amplified with the primers, pBYR2H-HSPter-F and pBYR2H-HSPter-R (Table S1) and inserted into *Xba*I-digested pBYR2H-EGFP, resulting in the plasmid pBYR2HH-EGFP (Fig. 1).

35Ster or NOSter was amplified with the primers, pBYR2T-35Ster-F and 35Ster-NOSter-R or 35Ster-NOSter-F and pBYR2TN-NOSter-R (Table S1), respectively. These PCR products were used as templates with primers, pBYR2T-35Ster-F and pBYR2TN-NOSter-R to produce 35Ster-NOSter sequences. This product was inserted into *Sal*I and *Xba*I-digested pBYR2H-EGFP, resulting in the plasmid pBYR2TN-EGFP (Fig. 1).

35Ster was amplified with the primers, pBYR2HS-35Ster-F and pBYR2HS-35Ster-R (Table S1), and inserted into *Xba*I-digested pBYR2H-EGFP or *Xba*I-digested pBYR2HS-EGFP, resulting in the plasmid pBYR2HT-EGFP or pBYR2HTS-EGFP (Fig. 1), respectively. 35Ster was also amplified with the primers, pBYR2T-35Ster-F and pBYR2HS-35Ster-R (Table S1), and then inserted into *Sal*I and *Xba*I-digested pBYR2H-EGFP, resulting in the plasmid pBYR2T-EGFP.

### Transient expression protocol in *N. benthamiana*, tomatoes, egg plants, hot peppers, melon, and a rose cultivar

The preparation of *Agrobacterium* and transient expression in *N. benthamiana* was performed as previously described^35^ with modifications. Briefly, the vectors described above and GFP_pICH18711 kindly provided by Dr. Victor Klimyuk (Icon Genetics GmbH) were transformed into *A. tumefaciens* GV3101. *A. tumefaciens* GV3101 harboring the binary vector was grown in L-broth media containing 10 mM MES (pH 5.6), 20 μM acetosyringone, 100 mg/L of kanamycin, 30 mg/L gentamycin, 30 mg/L of rifampin to the stationary phase at 28 °C. After centrifugation, *A. tumefaciens* was resuspended in the infiltration buffer (10 mM MgCl_2_, 10 mM MES (pH 5.6), 100 μM acetosyringone) to adjust OD_600_ = approximately 1. The suspension was infiltrated with a 1-mL syringe without a needle into the abaxial air spaces of 4-week-old leaves of *N. benthamiana*. The suspension was infiltrated into 4-week-old leaves of tomatoes, *Solanum lycopersicum* cv. ‘Micro-Tom’, 4-week-old leaves of eggplants, *Solanum melongena* cv. ‘Dewakonasu’, 4-week-old leaves of hot peppers, *Capsicum frutescens* L., 3-week-old leaves of melons, *Cucumis melo* cv. ‘Earl’s Favorite Harukei No. 3′, and petals of commercially produced orchids, *Phalaenopsis aphrodite* and the rose, *Rosa* sp. ‘Bonheur’. For infiltration into tomato fruits of *S. lycopersicum* cv. ‘M82’, a 1-mL syringe with a needle was used. Three days after infiltration, blue LED lamp was shed onto plants. GFP emission was detected with an ultraviolet absorbing filter (SC-52, Fujifilm).

### Transient expression protocol for lettuce

Preparation of *Agrobacterium tumefaciens* suspension and transient expression in lettuce was performed as previously described^36^ with modifications. *A. tumefaciens* GV3101 containing the binary vector was grown in modified YEB media (6 g/L of yeast extract, 5 g/L of tryptone, 5 g/L of sucrose, and 2 mM MgSO_4_) with antibiotics (100 mg/L of kanamycin, 30 mg/L gentamycin, and 30 mg/L of rifampin) for 2 days at 28 °C. Then, 2-day cultures were diluted 100 times in the same modified YEB with antibiotics, 10 mM MES (pH 5.6), and 20 μM acetosyringone, and grown for 18–24 h at 28 °C on a rotary shaker at 140 rpm. OD_595_ was reached to approximately 2. Then, 55 g/L of sucrose and 200 μM acetosyringone were added into the bacterial culture. The suspension was incubated for 1 h at 22 °C. After incubation at 22 °C, 2,4-dichlorophenoxyacetic acid and Tween-20 were added to the final concentrations of 100 μg/mL and 0.005%, respectively, and the suspension was subjected to vacuum-infiltration.

Red-leaf lettuce was obtained commercially from a local grocery store, rinsed with distilled water, and water was removed with paper towels. Then, the base of rinsed lettuce was placed on wet paper towels. The lettuce was covered with plastic wrap and incubated for one day at 24 °C. Before vacuum-infiltration, the lettuce was incubated with a blue LED light for more than 30 min. A 1.2-L portion of the *Agrobacterium* suspension was placed into a 2-L glass beaker inside a vacuum desiccator (Fig. S6A). The lettuce head was immersed into the suspension (Fig. S6B) and vacuum-infiltrated (29 in. Hg) for 20 min. After releasing the vacuum, the lettuce head was rinsed in water. The water was removed with paper towels. The base of rinsed lettuce was wrapped with wet paper towels and the lettuce was placed in a bowl (Fig. S6C). The lettuce was incompletely wrapped with plastic wrap, leaving some holes (Fig. S6D). The lettuce was incubated for 3–5 days at 24 °C under a 16-h light and 8-h dark photoperiod.

### Protein extraction and immunoblot analysis

Soluble protein was prepared as described previously with modification^37^. Briefly, Plant leaves (200 mg) were ground by bead beating using Cell Destroyer PS-1000 (Pro Sense, Inc., Tokyo, Japan) at 2,500 rpm for 10 s after freezing with liquid nitrogen. Then, 1 mL of lysis buffer [50 mM Tris-HCl, pH 8.0, 120 mM NaCl, 0.2 mM sodium orthovanadate, 100 mM NaF, 10% glycerol, 0.2% Triton X-100, 5 mM DTT, and 1× protein inhibitor cocktail (Nacalai Tesque, Inc., Kyoto, Japan)] was added. Powdered leaves and lysis buffer were mixed completely by bead beating and incubated on ice with shaking for 1 h. The samples were spun and liquid solution was used as a soluble protein extract at the concentration of 0.2 mg fresh weight (FW)/µL. To load a crude extract from 1 mg FW, 5 µL of sample solution was applied onto an SDS-PAGE gel. To load a crude extract from 0.2 mg FW, a crude extract was 5-fold diluted with lysis buffer (0.04 mg/µL) and, then, 5 µL of sample solution was applied. The gel was stained with Coomassie Brilliant Blue (CBB). The protein was also transferred onto a PVDF membrane (Amersham Hybond P PVDF, GE Healthcare). The blot was probed with anti-GFP antibody and detected using Luminata Forte Western HRP substrate (Millipore).

To compare expression level, each protein should be applied onto the same gel. Actually, the results can easily vary. Thus, we did three or more independent experiments. After staining with CBB, the gel was compared. We picked up the gel, in which the expression from pBYR2HS-EGFP was the least one among these gels, for Fig. 3A and calculate concentration of GFP expression by measuring band intensities using ImageJ software. Then, other gels containing similar GFP expression level from pBYR2HS-EGFP, compared to that in Fig. 3A, was picked up for calculation.

The whole gels of western blot analyses and CBB staining are provided in Supplemental Figures S1 to S5. Standard lines for calculation are also provided in Supplemental Figures S2 to S5.

## Electronic supplementary material

Supplementary information
