# Supplementary material for: Improvement of the transient expression system for production of recombinant proteins in plants
Source: Sci Rep. 2018 Mar 19;8:4755. doi: 10.1038/s41598-018-23024-y (PMC5859073; doi:10.1038/s41598-018-23024-y)
Supplement: Supplementary file 1 — Supplementary information [file 41598_2018_23024_MOESM1_ESM.pdf]

Supplementary Information for *Scientific Reports*

Improvement of the transient expression system for production of recombinant proteins in plants

Tsuyoshi Yamamoto[1§], Ken Hoshikawa[1§], Kentaro Ezura[1], Risa Okazawa[1], Satoshi Fujita[1], Miyo Takaoka[1], Hugh S. Mason[2], Hiroshi Ezura[1], Kenji Miura[1,*]

[1]Graduate School of Life and Environmental Sciences, University of Tsukuba, Tsukuba 305-8572, Japan
[2]Center for Immunotherapy, Vaccines and Virotherapy (CIVV), The Biodesign Institute at Arizona State University, Tempe, AZ 85287, USA
[§]These authors contributed equally to this work.

[*]Correspondence to email: miura.kenji.ga@u.tsukuba.ac.jp

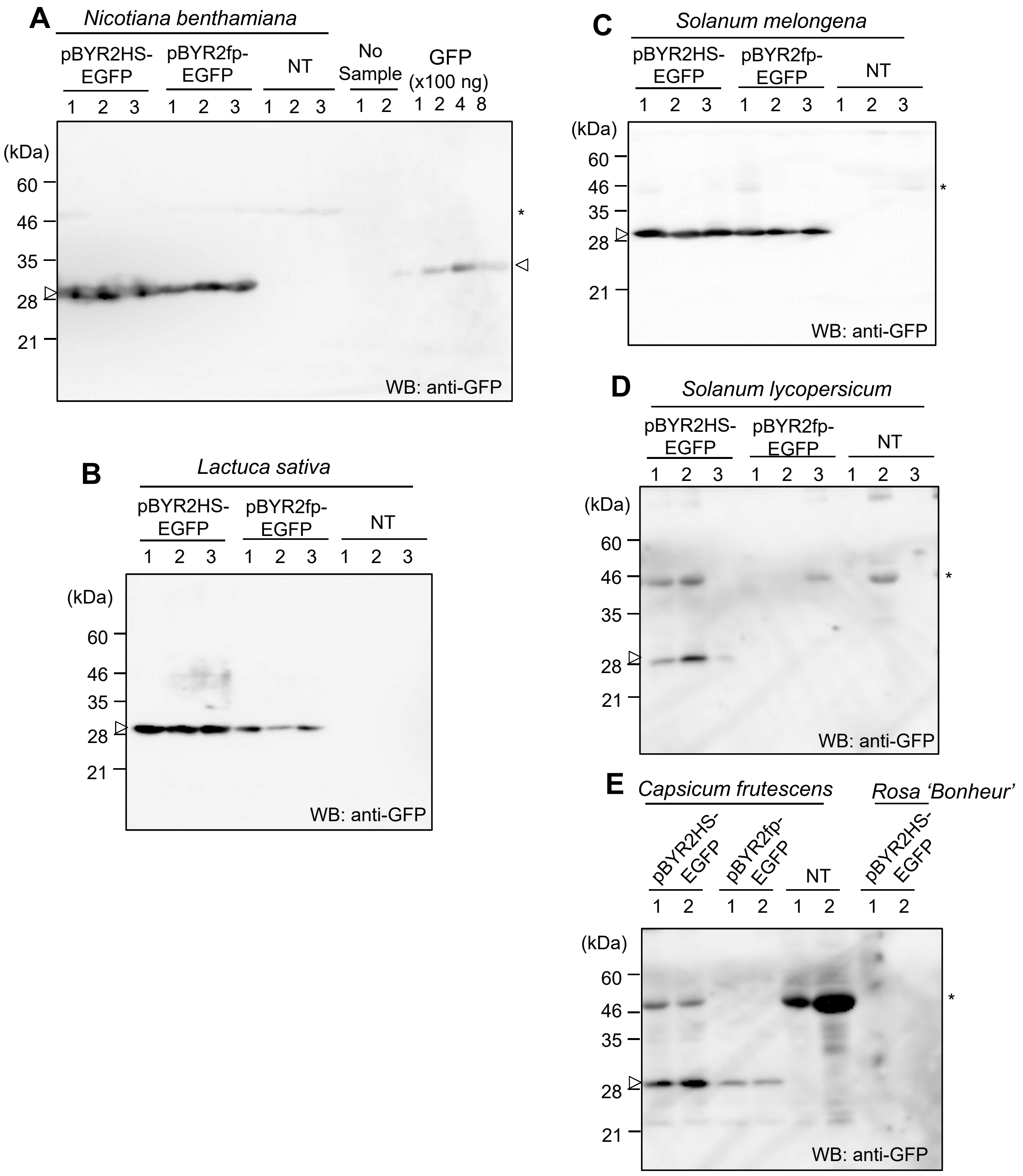

Figure S1. Full-length of blots with anti-GFP antibodies for Figure 2 are presented here. Blots from agroinfiltrated leaves of *N. benthamiana* (A), lettuce (*L. sativa*) (B), eggplants (*S. melongena*) (C), tomatoes (*S. lycopersicum*) (D), hot peppers (*C. frutescens*), and roses (*Rosa 'Bonheur'*) (E). Arrowheads indicate bands corresponding to GFP. Asterisks indicate non-specific bands.

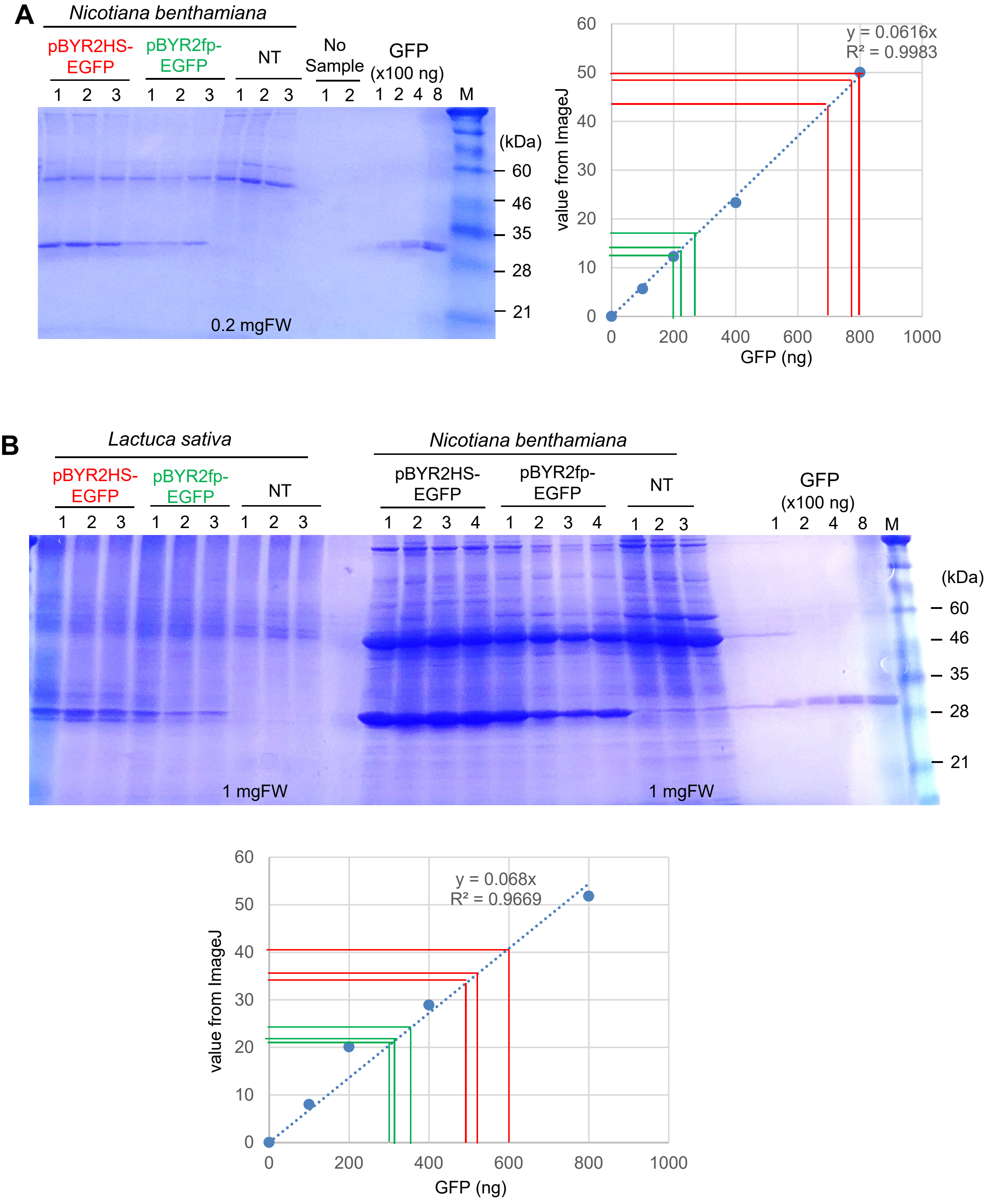
A
Nicotiana benthamiana
pBYR2HS-
EGFP
pBYR2fp-
EGFP
NT
No
Sample
GFP
(x100 ng)
1 2 3 1 2 3 1 2 3 1 2 1 2 4 8 M
(kDa)
60
46
35
28
21
0.2 mgFW
y = 0.0616x
R² = 0.9983
value from ImageJ
GFP (ng)
0 10 20 30 40 50 60
0 200 400 600 800 1000
B
Lactuca sativa
pBYR2HS-
EGFP
pBYR2fp-
EGFP
NT
1 2 3 1 2 3 1 2 3
Nicotiana benthamiana
pBYR2HS-
EGFP
pBYR2fp-
EGFP
NT
1 2 3 4 1 2 3 4 1 2 3
GFP
(x100 ng)
1 2 4 8 M
(kDa)
60
46
35
28
21
1 mgFW
1 mgFW
y = 0.068x
R² = 0.9669
value from ImageJ
GFP (ng)
0 10 20 30 40 50 60
0 200 400 600 800 1000

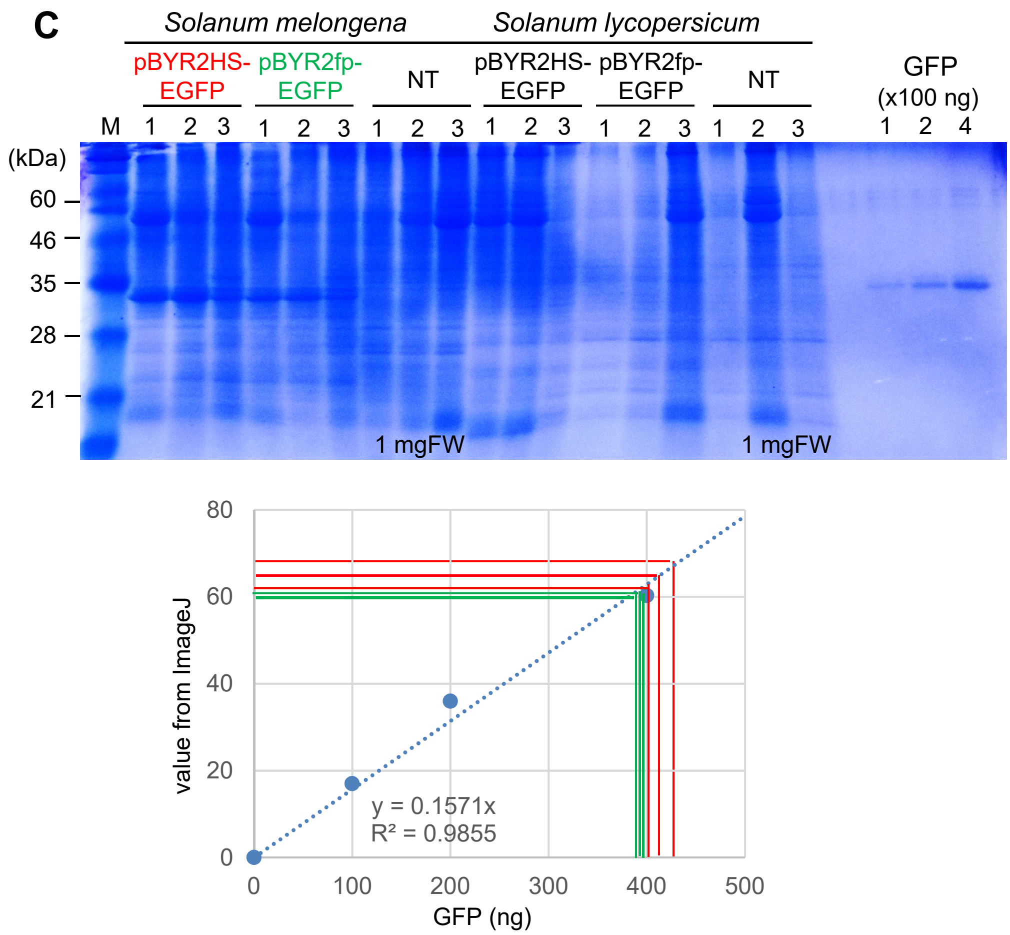

Figure S2. Whole gels for Figure 3A-3C stained with CBB are presented here. In each gels, GFP standards were loaded. To compare concentrations, GFP standards are needed to be loaded in the same gels. Value of the GFP band and background near the GFP band calculated by ImageJ were measured and the difference was used for value of the band. The value of standard GFP band was plotted onto graph, and standard line was calculated. Red lines and green lines indicate the value of GFP band from pBYR2HS-EGFP and pBYR2fp-EGFP, respectively. (A) Total soluble protein from 0.2 mgFW of *N. benthamiana* was loaded onto an SDS-PAGE gel. Standard line was calculated from concentration of GFP and the value of GFP band measured from ImageJ. (B) Total soluble protein from 1 mgFW of lettuce (*Lactuca sativa*) and *N. benthamiana* was loaded onto an SDS-PAGE gel. Because too much protein band was observed in *N. benthamiana*, diluted solution was loaded as shown in (A). Standard line is provided for calculation of expression levels in lettuces. (C) Total soluble protein from 1 mgFW of eggplant (*Solanum melongena*) and tomato (*Solanum lycopersicum*) was loaded onto an SDS-PAGE gel. Because no obvious GFP band was detected in the soluble protein from tomato, concentration of GFP was not able to be calculated. Only expression levels in eggplants were calculated.

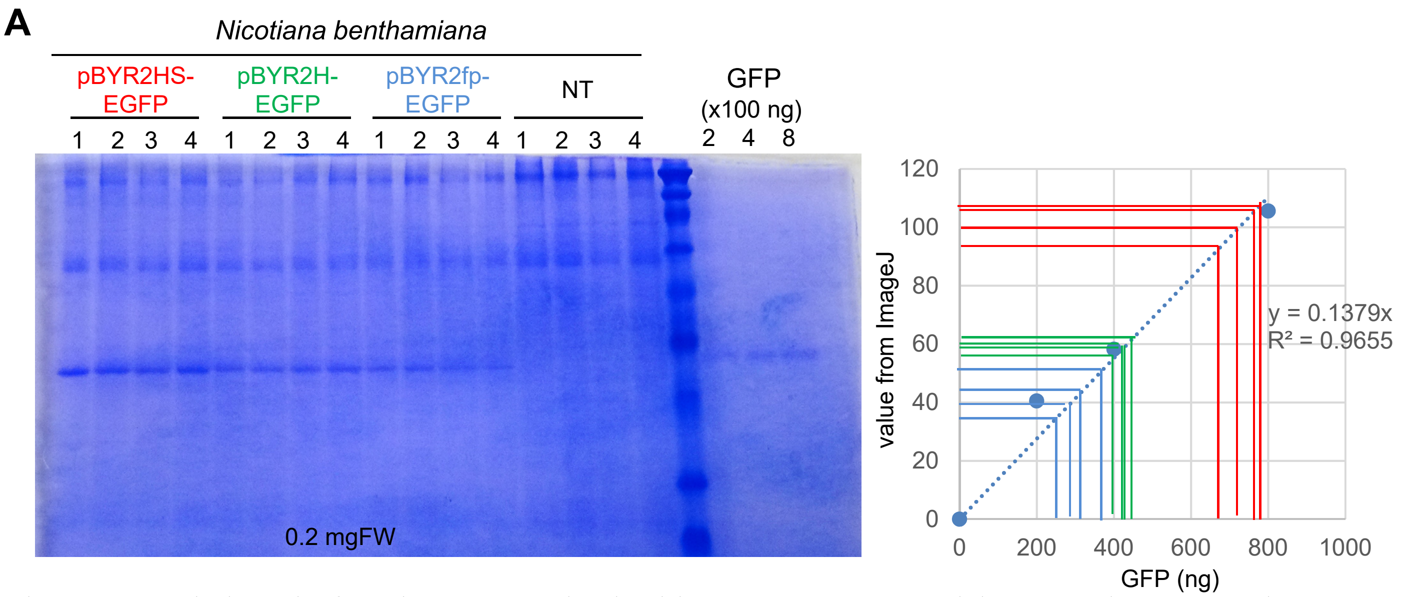

Figure S3. Whole gels for Figure 4F stained with CBB are presented here. In the same gels, GFP standards were loaded to compare concentrations. Value of the GFP band and background near the GFP band calculated by ImageJ were measured and the difference was used for value of the band. The value of standard GFP band was plotted onto graph, and standard line was calculated. Red lines, green lines, and blue lines indicate the value of GFP band from pBYR2HS-EGFP, pBYR2H-EGFP, and pBYR2fp-EGFP, respectively. Total soluble protein from 0.2 mgFW of *N. benthamiana* was loaded onto an SDS-PAGE gel. Standard line was calculated from concentration of GFP and the value of GFP band measured from ImageJ.

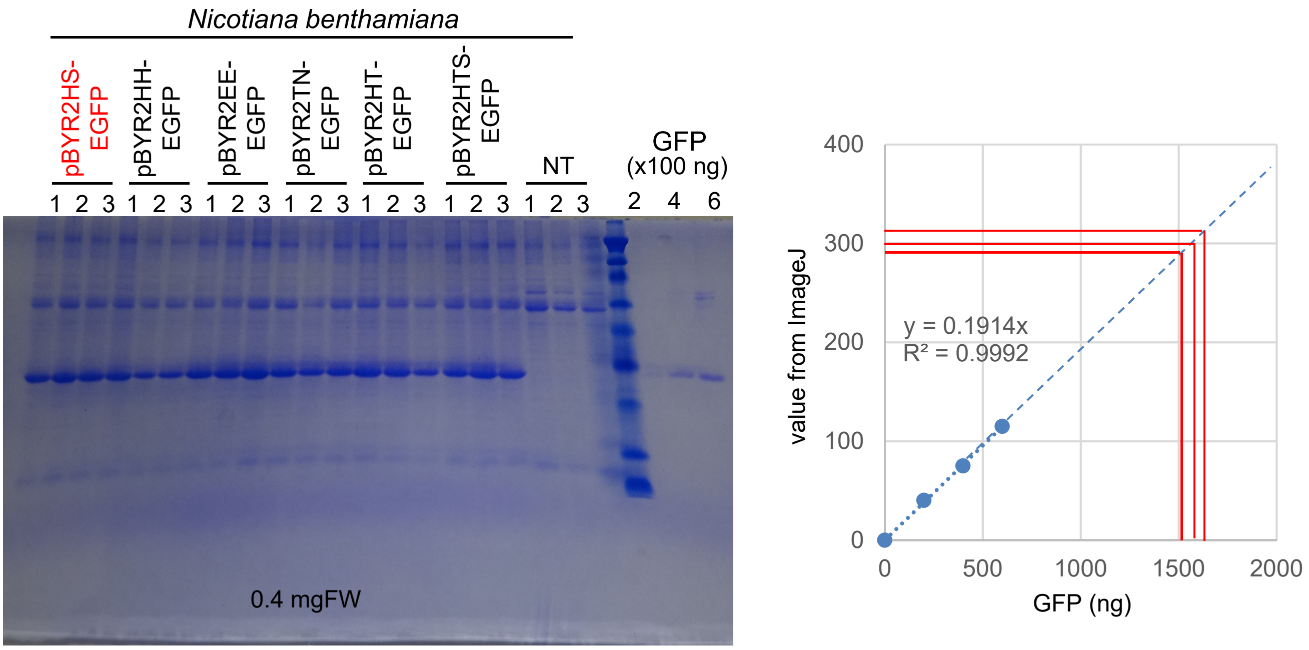

Figure S4. Whole gels for Figure 4H stained with CBB are presented here. In the same gels, GFP standards were loaded to compare concentrations. Value of the GFP band and background near the GFP band calculated by ImageJ were measured and the difference was used for value of the band. The value of standard GFP band was plotted onto graph, and standard line was calculated. Red lines indicate the value of GFP band from pBYR2HS-EGFP. Total soluble protein from 0.4 mgFW of *N. benthamiana* was loaded onto an SDS-PAGE gel. Standard line was calculated from concentration of GFP and the value of GFP band measured from ImageJ.

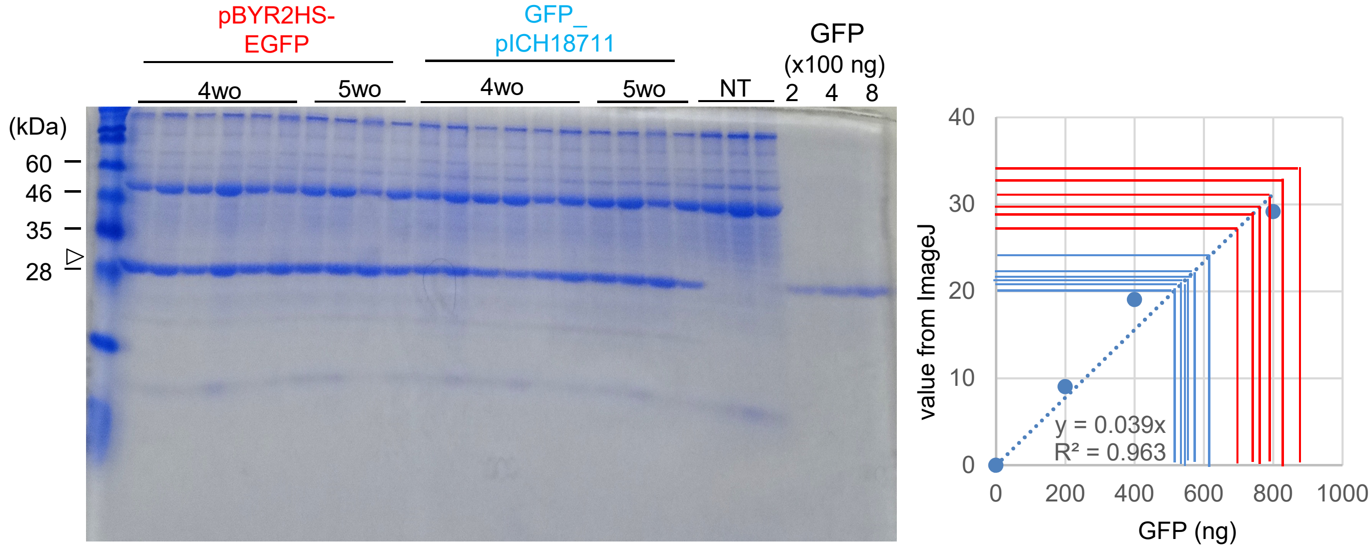

Figure S5. Whole gels for Figure 4F stained with CBB are presented here. In the same gels, GFP standards were loaded to compare concentrations. Value of the GFP band and background near the GFP band calculated by ImageJ were measured and the difference was used for value of the band. The value of standard GFP band was plotted onto graph, and standard line was calculated. Red lines, green lines, and blue lines indicate the value of GFP band from pBYR2HS-EGFP, pBYR2H-EGFP, and pBYR2fp-EGFP, respectively. Total soluble protein from 0.2 mgFW of *N. benthamiana* was loaded onto an SDS-PAGE gel. Standard line was calculated from concentration of GFP and the value of GFP band measured from ImageJ.

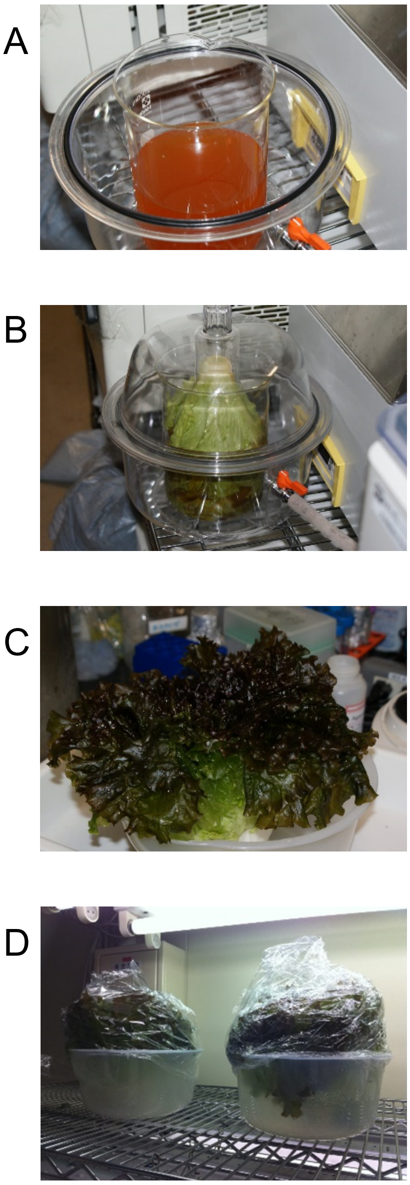

Figure S6. Vacuum-infiltration of lettuce. *Agrobacterium tumefaciens* with the binary vector was grown for 18–24 h at 28° C in modified YEB media with 10mM MES (pH 5.6), 20 μM acetosyringone, and antibiotics. After incubation, *Agrobacterium* media was transferred to a 2-L glass beaker (A). (B) The lettuce head was immersed into the *Agrobacterium* suspension and infiltrated with a vacuum for 20 min. (C) After infiltration with the vacuum, the lettuce head was rinsed in water and the water was removed with paper towels. The base of rinsed lettuce was put on wet paper towels. Then, the lettuce was put in a bowl. (D) The lettuce was wrapped with plastic wrap and incubated for 3–5 days.
